# The Evolution of Living Kidney Donation and Transplantation in Older Adults

**DOI:** 10.1111/jgs.13832

**Published:** 2015-12-11

**Authors:** Theresa A. Rowe, Jill Huded, Lisa McElroy, Daniela P. Ladner, Lee A. Lindquist

**Affiliations:** * Division of General Internal Medicine and Geriatrics, Comprehensive Transplant Center, Feinberg School of Medicine, Northwestern University, Chicago, Illinois; † Northwestern University Transplant Outcomes Research Collaborative, Comprehensive Transplant Center, Feinberg School of Medicine, Northwestern University, Chicago, Illinois

**Keywords:** kidney transplantation, living donors, organ donation

## Abstract

Kidney transplantation is a good option for adults aged 65 and older with end-stage renal disease because it has been shown to reduce morbidity and mortality, improve quality of life, and is more cost-effective than other renal replacement options. However, older age has been a deterrent to access to the deceased donor waiting list, and individuals aged 65 and older have a lower probability of being referred to and listed for transplantation compared to younger adults. Because the deceased organ supply is limited, living donor kidney transplantation offers an effective alternative for older adults facing long waiting times for cadaveric organs. This article describes the evolution of living kidney donation and transplantation in older adults over 15 years using the Organ Procurement and Transplantation Network/Scientific Registry of Transplant Recipients database. Between 1997 and 2011, 28,034 kidney transplantations were performed in adults aged 65 and older. Living-donor and cadaveric kidney transplantation increased in older adults over the 15-year period. Offspring are the most common living donors in this age group, followed by unrelated donors (e.g., friends), whereas the most common donors in younger transplant recipients are spouses, siblings, and parents. The number of living kidney donors aged 65 and older is slowly increasing, although the total number of transplants in this age group remains low. The expansion of living-donor kidney transplantation in the aging population may offer a solution for organ shortage and thereby improve the quality of life of older adults. More research is needed to understand the older donor–recipient relationship and barriers to transplantation in this population.

The number of adults aged 65 and older with end-stage renal disease (ESRD) is growing, with new cases estimated to be more than 55,000 per year in the United States.^1^ As the population ages, this number is expected to rise, making efforts to improve the management of ESRD in older adults increasingly important. Kidney transplantation is a good option for many older adults with ESRD, because it has been shown to reduce morbidity and mortality, improve quality of life and is more cost effective than other renal replacement options, such as hemodialysis.^2,3^ Over the past decade, kidney graft survival has increased and overall mortality has decreased substantially in older transplant recipients, even in those aged 80 and older.^4^ Furthermore, quality of life in older adults after kidney transplantation has been shown to be better than in younger transplant recipients and equal to that of the general population.^5^ Nevertheless, older age has been a deterrent to access to the deceased donor waiting list, and older adults have a lower probability of being referred and listed for kidney transplant, even in the absence of an absolute contraindication, than younger adults. In a group of adults who were recently initiated on hemodialysis in Maryland, those aged 65 and older were less likely to have even discussed transplantation with a medical professional, friends, or family (44.5%) than those younger than 65 (74.8%).^6^

Several barriers have been described that may discourage older adults from seeking renal transplantation. The evaluation process for kidney transplantation is complex and requires multiple physician appointments and testing (e.g., coronary angiogram, colonoscopy) before being listed for transplantation. Although this evaluation is compulsory to assess eligibility, long waiting times for these procedures, transportation challenges, and the overall complexity of the process can deter older adults from seeking transplantation.^7,8^ Individuals aged 65 and older have a higher prevalence of comorbidities (e.g., coronary artery disease, congestive heart failure, vascular disease) and may not think they are not eligible for transplantation because of chronic health conditions,^7^ but new risk-prediction models have been developed that may help identify older adults who would most benefit from kidney transplantation, showing that up to 75% of older adults with ESRD have excellent posttransplant outcomes.^9^ Even so, ethical concerns that older adults may be taking kidneys from younger, “healthier” adults have been raised, and new policies and regulatory procedures focused on allocating kidneys to individuals who obtain the greatest survival benefit from transplantation have made it difficult for older adults to undergo kidney transplantation.^10^ Despite these barriers, the number of older adults considering renal transplantation continues to increase, with more than 23,000 adults aged 65 and older currently on the waiting list for kidney transplant.^1^ Because deceased organ supply is limited, living donor kidney transplant (LDKT) offers an alternative that can be an efficient solution for older adults burdened with long waiting times for cadaveric organs. However, little is known about the relationships between living kidney donors and the older population and how these relationships compare with those of younger adults. Understanding who provides living kidney donations to older adults is important because the need for available organs is dramatically greater in this population. The aim of this article is to describe the evolution of kidney donations in individuals aged 65 and older over the past 15 years.

## EVOLUTION OF KIDNEY DONATIONS

This was a secondary data analysis using the Organ Procurement and Transplantation Network/Scientific Registry of Transplant Recipients database, which includes information on all wait-listed transplant candidates, recipients, and donors in the United States. Individuals aged 18 and older who received a kidney transplant from January 1997 through December 2011 were included. Individuals for whom donor information was not available were excluded (<1%). Statistical analysis was performed using SPSS version 17.0 (SPSS Inc., Chicago, IL). Kidney transplant rates were calculated according to age group (18–34, 35–49, 50–64, ≥ 65) for each year from 1997 to 2011. Frequency of each donor type was then calculated for each category and age group.

### Frequency of Kidney Transplantation

**Figure 1.**
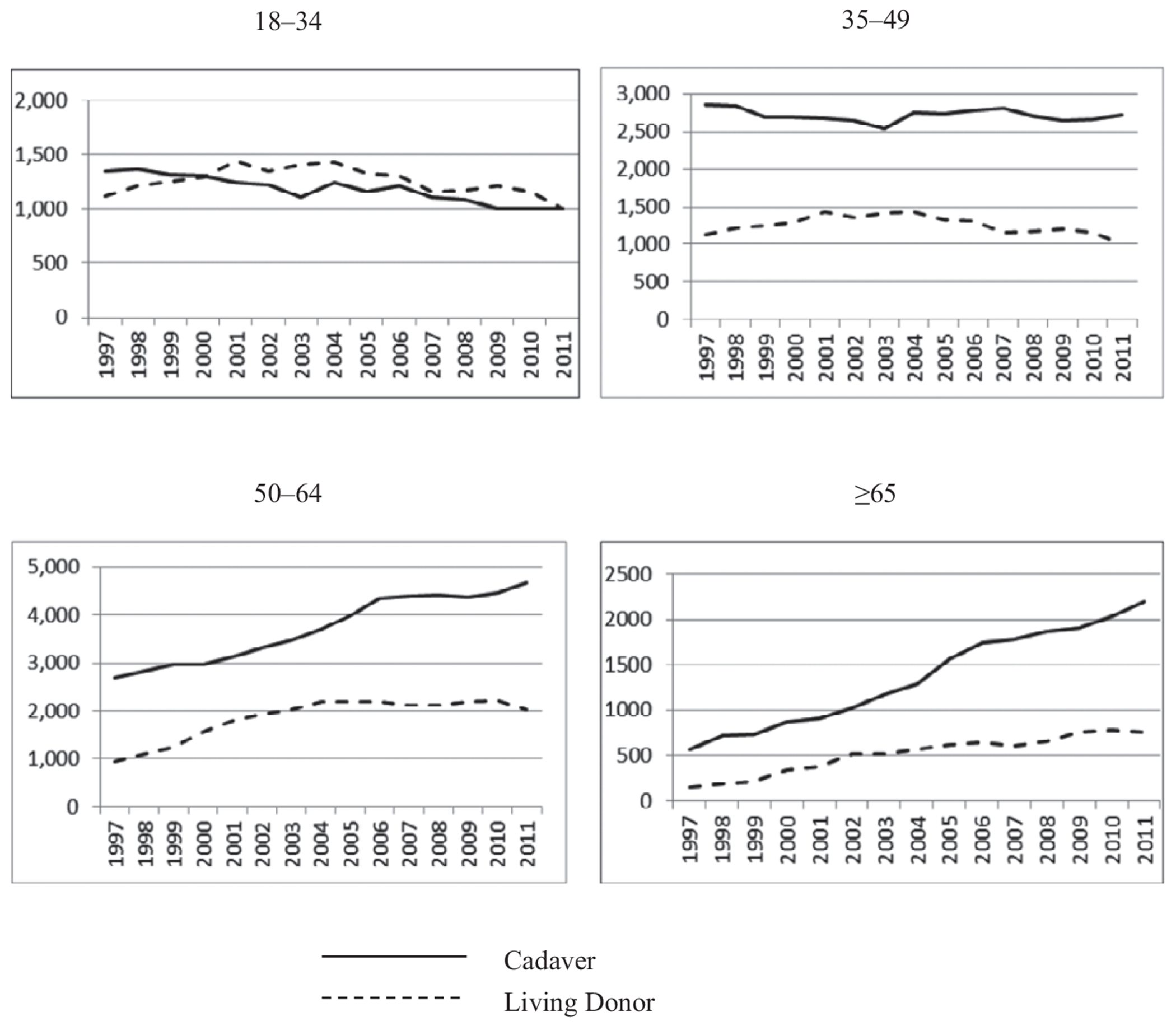
Frequency of donor type according to year and age group.

From 1997 to 2011, 28,034 adults aged 65 and older underwent kidney transplantation. In 2011, those who received a kidney included 2,948 individuals aged 65 and older, 6,681 aged 50 to 64, 4,410 aged 35 to 49, and 2,010 aged 18 to 34. There has been continued growth in the number of kidneys transplanted in adults aged 65 and older (Figure 1).

### Donor Types of Kidneys Transplanted into Individuals Aged 65 and Older

Over the past 15 years, cadaveric transplantation in older adults has grown significantly, and although donations from other sources have also increased, the absolute number of LDKTs in older adults remains low. In individuals aged 65 and older receiving a kidney transplantation in 2011, the most common donor was cadaveric, increasing from 571 in 1997 to 2,197 in 2011, followed by offspring, increasing from 92 in 1997 to 324 in 2011, and unrelated donor, increasing from 18 in 1997 to 168 in 2011. Other types of donated kidneys in 2011 included spouse or life partner (n = 73), unrelated paired exchange (n = 70), sibling (n = 49), other blood relative (n = 50), and anonymous donation (n = 17). In younger individuals, cadaveric donors were still the most common, although sibling donors replaced offspring as the second most common donor in those younger than 50. Table 1 illustrates the most common donor types according to age group from 1997 to 2011.

### Kidneys from Living Donors Aged 65 and Older

Over the past 15 years, 1,054 adults 65 and older have been living kidney donors. The number of living adult donors aged 65 and older has increased from 2005 (n = 58) to 2010 (n = 119) but decreased in 2011 (n = 95). From 2000 to 2007, the number of male and female donors aged 65 and older were similar (272 female, 232 male). From 2008 to 2011, women were twice as likely to be living donors as men (273 women, 137 men).

## DISCUSSION

This article describes the evolution of kidney donations to older adults over a 15-year period. Cadavers remain the most common donor source of kidneys for transplant in older adults, and there has been significant growth over time. Although LDKT has also increased, the total number of LDKTs in the older adult population remains lower than in younger groups. Offspring are the most common living donors in individuals aged 65 and older, followed by unrelated donors (e.g., friends), whereas the most common donors in younger transplant recipients are spouses, siblings, and parents. The total number of living kidney donors aged 65 and older, although increasing, is still low.

### Deceased Donor Transplantation

Although older adults have received a larger share of deceased donor kidneys over the past 15 years, the number of deceased donors has been stagnant, ultimately leading to longer waiting times for older adults. Older adults on average spend longer than 1,500 days on the deceased donor waiting list, compared with less than 1,000 days for those aged 18 to 34.^11^ The incidence of death while on the waiting list rises with age, and individuals aged 65 and older have mortality of 7.5 deaths per 100 waitlist years, versus 2.2 deaths per 100 waitlist years for those aged 18 to 34.^12^ Greater use of deceased donor kidneys has been achieved through the use of more-marginal deceased donors (e.g., aged ≥65, with a history of hypertension), and these extended criteria donations (ECD) have been found to reduce the risks of waiting list mortality by 25%.^13^ Furthermore, the risks of receiving an extended criteria organ have been shown to outweigh the risks associated with extended dialysis in older adults.^14^ Even with the expansion of deceased donor kidneys using ECD, the number of available deceased donor organs is unlikely to meet the growing demand for kidneys of older adults with ESRD seeking kidney transplantation.^15^

### LKDT in Older Adults

One option to expand the available organ pool for older adults is LDKT. LDKT is associated with better survival in all age groups compared to deceased donor transplantation.^16^ Three-year survival with LDKT is greater than 80%, compared with 70% for cadaveric transplants, with a hypothesis that the difference in survival is due to damage to cadaveric grafts before removal from the donor.^17^ Similar if not better survival rates have been seen in adults aged 80 and older, with 3-year survival for LDKT of 87%, versus 70% for deceased donor transplants, suggesting better outcomes even in very old adults.^4^ Although greater comorbidity has been associated with poorer graft survival in deceased donor transplants, the effect of increasing comorbidity appears to be less in older adults undergoing LDKT.^18^ Despite the benefits associated with LDKT, only a small fraction of kidney transplants in older adults are from living donors, and an even smaller percentage from donors aged 65 and older.^19^ This article describes how living donor types change over the course of a person’s lifespan and how changing relationships may affect living kidney donation. As adults marry and have children, these relationships play a larger role in organ donation, and offspring become the most common source of living kidney transplant in adults aged 65 and older. There has also been a slight increase in unrelated donor (e.g., friends) and unrelated paired exchanged donors over the past 15 years. As adults get older, siblings and spouses as donors begin to decrease, potentially because of comorbidities in these aging groups, but many older adults (e.g., siblings and spouses) may still be living organ donors, despite their increasing age,^17^ and efforts to improve older living donor directed organs (donors aged ≥65) may offer an effective solution for increasing the number of available living donor kidneys in this population. Although kidney donors aged 65 and older have been increasing, this trend has remained relatively flat over several years. Older donor age has been associated with lower graft survival rates^20^ and with higher rehospitalization rates after transplantation, but many older adults can still benefit from transplantation of organs from older living donors, and studies have shown that survival in individuals who receive an older live donor kidney is as good or better compared to those who received a cadaveric organ.^19^ Additionally, aligning older living kidney donors with older recipients has resulted in graft survival outcomes similar to those of young-to-young allocations.^21^ The common factor in all studies is the importance of donor selection, and consensus guidelines for elderly organ donor selection are needed.

### Barriers to Living Kidney Donation in Older Adults

Low rates of LDKT in older adults, despite known good outcomes, suggests there are factors that may prevent older adults from donating and accepting a kidney. As the relationships of living donors change throughout a person’s lifetime, barriers to LKDT also change and are likely to be different from those in younger individuals. From a living donor perspective, older adults and clinicians may incorrectly assume they are not potential organ donors because of their advanced age, even though older donors have the potential to provide excellent kidneys to older recipients. A recent study found that, after adjustment for age, living donor kidneys from older adults had better renal function, as measured using glomerular filtration rate, than kidneys from younger donors.^19^ In older adults who are healthy enough to donate an organ, undergoing elective surgery may be daunting when faced with perceived risk and uncertainty of the future. Although living kidney donation has been associated with greater risk of ESRD than in healthy individuals who are not donors, the overall risk appears to be lower than in the general population.^22^ Similarly, potential transplantation recipients may not want loved ones—aged spouses, friends, siblings—to undergo the risk of surgery or potential future problems. In an ethnographic study, fewer than half of individuals who were offered a living kidney donation accepted it. Living donor health was a prominent concern in those individuals.^23^ As surgical techniques advance, living kidney donations pose a lower surgical risk to potential donors, even in older adults. Although median length of hospital stay may be longer in older adults, intraoperative and postoperative complications do not vary according to donor age.^24^ Mortality was not significantly greater in a cohort of live kidney donors than in nondonors.^25^ Postoperative quality of life in donors aged 60 and older is comparable to that of younger donors, with dimensions of bodily pain and vitality in favor of the elderly cohort.^26^ Furthermore, a recent study found that, in the subset of prior donors who developed ESRD, median time to kidney transplantation was significantly lower than in matched nondonors (145 vs 1,607 days), and these individuals had lower mortality than nondonors (hazard ratio = 0.19, 95% confidence interval = 0.08–0.46, *P* < .001), although the median age in that study was 52, so its applicability to older adults is unknown.^27^ Knowing how and when to initiate a discussion on living donation is another significant barrier to proceeding with renal transplantation in all age groups. In a questionnaire given to adults on the deceased donor list, not knowing how to ask someone to donate a kidney was the most frequently reported barrier, occurring in 71% of participants in the study.^28^ This may be particularly burdensome for older adults who are asking their own offspring or aged spouse to undergo a major operation. Although barriers have been described that may preclude potential recipients from LDKT, few studies have focused specifically on older adults. Further studies evaluating perceived limitations to LDKT specifically in adults aged 65 and older are needed.

### Conclusions

As the population continues to age, the number of older adults living with ESRD will continue to grow. This article describes how living donor types change over the course of a person’s life span and how changing relationships may affect living kidney donation. LKDT has consistently been associated with better outcomes than deceased-donor transplantation in older adults with ESRD and to extended time on dialysis. Although older adults should be thoroughly screened for comorbidities that would exclude them from transplantation,^29^ age alone should not discourage healthcare providers from referring people for possible transplantation. Furthermore, age should not prevent older adults from being considered for living kidney donation. Multiple factors influence the decision to donate and accept a living donor kidney, and further research is needed to better understand the older donor–recipient relationship and barriers to transplantation. LDKT rates may improve with educational programs targeted at transplant recipients and potential organ donors about how to initiate the living donation process, and studies to evaluate such educational programs in older adults are needed.^30^ A safe solution to alleviate the kidney shortage for the aging population with ESRD is available, and with better awareness and understanding of LKDT, quality of life for thousands of older adults may be improved.

## Figures and Tables

**Table 1.** Most-Common Donor Type According to Age Group over Past 15 Years

18–34	35–49	50–64	≥65
**n**			
Cadaveric, 17,730	Cadaveric, 40,741	Cadaveric, 55,710	Cadaveric, 20,344
Sibling, 7,016	Sibling, 11,582	Offspring, 8,992	Offspring, 4,017
Parent, 5,454	Unrelated directed, 5,602	Sibling, 5,769	Unrelated directed, 1,496
Unrelated directed, 2,802	Spouse or life partner, 4,384	Unrelated directed, 5,697	Spouse or life partner, 775
Other relative, 1,759	Offspring, 2,024	Spouse or life partner, 4,329	Other relative, 557
Spouse or life partner, 1,258	Other relative, 1,917	Other relative, 1,679	Sibling, 482
